# The effect of *Aspergillus niger* as a dietary supplement on blood parameters, intestinal morphology, and gut microflora in Haidong chicks reared in a high altitude environment

**DOI:** 10.14202/vetworld.2020.2209-2215

**Published:** 2020-10-23

**Authors:** Hao Lin, Baoan Ding, Lingyun Chen, Zhenming Zhang, Hailian He, Jingge Wang, Xiezhong Wang, Licheng Zhang, Xiaoming Ni, Baldassare Fronte

**Affiliations:** 1 State Key Laboratory of Plateau Ecology and Agriculture, College of Agriculture and Animal Husbandry, Qinghai University, Xining 810016, China; 2 Qinghai Animal Disease Control Center, Xining 810001, China; 3 Department of Science Veterinary, University of Pisa, Viale delle Piagge 2, 56124, Pisa, Italy

**Keywords:** *Aspergillus niger*, gut microflora, Haidong chicks, intestinal morphology, probiotic

## Abstract

**Aim:**

The effects of the inclusion of *Aspergillus niger* in the diet of Haidong chicks reared in the Qing-Zang high altitude area (China) under hypoxic conditions.

**Materials and Methods:**

A total of 720 Haidong chicks were randomly divided into six groups and fed diets supplemented with 0%, 0.5%, 0.75%, 1.0%, 1.25%, and 1.5% of *A. niger* to determine blood parameters, intestinal morphology, and gut microflora in Haidong chicks reared in a high altitude environment.

**Results:**

Packed cell volume (PCV), red blood cell (RBC) count, white blood cell count, and hemoglobin concentration increased in the groups fed diets containing *A. niger*. The administration of *A. niger* in 1.0% and 1.25% significantly decreased the concentration of *Escherichia coli* in the cecum, while the concentration of *Bifidobacterium* and *Lactobacillus* in the cecum and ileum was increased in the treated groups. When compared to the control groups, villi height, crypt depth, and goblet cell density in the intestine was raised, in general, in the groups treated with *A. niger*.

**Conclusion:**

These findings suggest that 1.25% *A. niger* as dietary supplement may improve the resistance to ascites among birds reared under hypoxic conditions.

## Introduction

Probiotics are defined as viable microorganisms that in sufficient amounts on reaching the intestine in an active state can exert positive health effects [1]. Several investigations have investigated the effects of probiotics such as *Aspergillus* spp. on animal health; in particular, they reported that poultry growth is promoted by supplementing feeds with probiotic or ­prebiotics [2-6]. Yet, other studies have stated that supplementation of probiotics has no effect on the health of broiler chicks [7-9].

It has been suggested that probiotics act to stimulate the production of antimicrobial substances and organic acids, protection of the villi, and absorptive surfaces against toxins produced by pathogens, as well as the stimulation of the immune system [10,11]. Moreover, recent papers infer that probiotics can lower serum CH levels and increase packed cell volume (PCV), hemoglobin (Hb), and red blood cell (RBC) values [5]. Ascites is a metabolic disorder and is a multi-factorial syndrome caused by the interaction between genetic, physiological, environmental, and management factors [12]. It is characterized by hypoxemia, increased workload of the cardiopulmonary system, central venous congestion, an excessive accumulation of fluid in the body coelomic cavities [13], hypertrophy of the right ventricle (RV) and a flaccid heart [14], and finally death. A high incidence of ascites can occur when the broilers are reared at high altitudes under hypoxic conditions [15]. It is estimated that 5% of broilers and 20% of roaster birds perish due to ascites and this results in significant economic loss. To this end, Solis de los Santos *et al*. [16] infer that broilers fed prebiotics under hypoxic conditions showed 23% lower incidence of ascites. These results are encouraging as they offer a potential solution to the problem of rearing fowl under high altitude conditions.

We investigated the effects of the probiotic *Aspergillus niger* as a dietary supplement on chicks reared in the Qing-zang high altitude area (China), accordingly. Specifically, we assessed the blood parameters, intestinal function, and overall intestinal morphology in birds affected with ascites. We also evaluated the histology and microflora of broiler chickens impacted with this condition.

## Materials and Methods

### Ethical approval

All procedures were in compliance with the national laws and regulations for animal experimentation and performed in accordance with the guiding principles of Qinghai University Animal Care Committee for the care and use of experimental animals.

### Study period and location

The experiment was carried-out in Xueling Haidong Chicken Breeding Farm, Huzhu County, Qinghai, China, from December 2018 to June 2019.

### Bird management and treatment

All experiments were conducted in Huzhu County in Qing-Zang plateau in northwestern China, at an altitude range of 2500-3000 m above sea level. A total of 720, 1-day-old commercial male Haidong chicks were randomly selected and divided into six treatments, in six replicates (with 20 chicks). Treatments consisted of adding 0%, 0.5%, 0.75%, 1.0%, 1.25%, and 1.5%, of *A. niger* (hereafter referred to as C, A1, A2, A3, A4, and A5, respectively, *A. niger* from filamentous fungus (1 × 10^9^ spores/g) to the feed of experimental animals. Diets (mash) were formulated to meet the National Research Council nutrient requirements. The chicks were fed experimental diets (Table-1) until 42 days of age (d). The ingredients and chemical compositions of the diets were analyzed using AOAC (1990) procedures. The chicks were placed in wire-floored, stainless-steel cages and kept indoors between 30°C and 32°C from 1 to 14 days, and at 24°C and 26°C from 15 to 21 days. Light was provided continuously with incandescent bulbs throughout the experimental period (5 lux). Feed and water were provided *ad libitum*.

**Table 1:** Ingredients and chemical analyses of broiler chicken diets.

Ingredients (%)	C	*Aspergillus niger*				

		A1	A2	A3	A4	A5
Yellow Corn	52.77	52.77	52.77	52.77	52.77	52.77
Soybean meal (46%)	36.0	36.0	36.0	36.0	36.0	36.0
Wheat bran	3.0	2.5	2.25	2	1.75	1.5
Soybean oil	3.6	3.6	3.6	3.6	3.6	3.6
Limestone	1.16	1.16	1.16	1.16	1.16	1.16
*Aspergillus niger*	0	0.5	0.75	1.0	1.25	1.5
DL-Methionine	0.2	0.2	0.2	0.2	0.2	0.2
L-Lysine	0.02	0.02	0.02	0.02	0.02	0.02
Vitamin-mineral Premix	1.0	1.0	1.0	1.0	1.0	1.0
Sodium chloride	0.3	0.3	0.3	0.3	0.3	0.3
Dicalcium phosphate	1.95	1.95	1.95	1.95	1.95	1.95
Calculated						
ME (kcal/kg)	2988	2986	2984	2982	2980	2981
Dry matter (%)	89.5	89.7	89.6	89.5	89.8	89.6
CP (%)	21.6	21.4	21.4	21.3	21.3	21.4
Fat (%)	2.4	2.4	2.4	2.4	2.4	2.4
Methionine	0.64	0.64	0.64	0.64	0.64	0.64
Lysine	1.24	1.23	1.23	1.24	1.23	1.23
Ca (%)	0.98	0.98	0.98	0.98	0.98	0.98
Av. P (%)	0.49	0.49	0.49	0.49	0.49	0.49

Supplied per kilogram of diet: Vitamin A, 15000 IU; Vitamin D3, 3000 IU; Vitamin E, 26 IU; Vitamin K, 2.5 mg; Vitamin B1, 2.1 mg; Vitamin B2, 5 mg; Vitamin B6, 3 mg; Vitamin B12, 6 mg; folic acid, 1 mg; biotin, 50 μg; niacin, 24 mg; choline chloride, 50 mg, manganese, 45 mg, iron, 83 mg; zinc, 10 mg, copper, 0.26 mg; iodine, 1.5 mg; selenium, 1.2 mg

### Blood analysis

One bird from each replicate was randomly chosen and blood samples taken from the brachial vein on day 42 after 6 h of fasting. The blood samples were collected into both heparinized test tubes and non-heparinized tubes; the heparinized blood samples were centrifuged (2000 rpm for 10 min) for serum separation while the blood in plain, non-heparinized tubes were stored at −20°C until assayed. The following blood parameters were measured: Cholesterol (CH), triglycerides (TG), high-density lipoprotein (HDL), and low-density lipoprotein (LDL) PCV, RBC count, white blood cell (WBC) count, and Hb. The index of PCV, RBC, and WBC was determined with the use of an automated hematology analyzer (Sysmex KX-21, Kobe, Japan). Hb concentration was calculated using the cyanmethemoglobin method. The concentration of CH, TG, HDL, and LDL was estimated according to the procedures outlined by the manufacturer of the diagnostic kits (BioSystems, S.A. Barcelona, Spain) and spectrophotometer apparatus.

### Histological analysis of the intestinal morphology

One bird from each replicate was randomly selected on day 42, and sacrificed by cervical dislocation. Segments of approximately 5 cm of duodenum (midpoint of the pancreatic loop), jejunum (midpoint of jejunum), and ileum (after Meckel’s diverticulum) were obtained before removing the entire intestinal tract. The samples were fixed in fresh 4% buffered formalin solution for 24 h and processed for routine paraffin embedding. Serial 4 μm transverse sections were cut and stained (routine Hematoxylin and Eosin staining) for morphological evaluation. Goblet cells containing acidic and neutral mucin were counted by staining 4 μm sections with alcian blue (AB) pH 2.5 + periodic acid-Schiff reagent (PAS). Sections were examined using a light microscope (Leitz, Diaplan) connected to a PC via a Nikon digital system (Digital Sight DS-U1). Images were acquired using the NIS-Elements F version 2.10 software (Nikon Corporation, Japan) per transverse section of small intestine. Measurements were made using the ImageJ 1.37V software (National Institutes of Health, Bethesda, MD, USA) package. Ten well-oriented and intact crypt-villus units of each slide were measured in triplicate. Villi height was defined as the distance from villus tip to crypt junction. Crypt depth was defined as the depth of the invagination between adjacent villi. Muscle thickness was measured from the junction between the submucosal and muscular layers to the muscular layer and the tunica serosa. Intestinal wall thickness was defined as the distance from intestinal external subserosa to the junction muscular layer and submucosa. The number of AB/PAS-positive cells along the villi was expressed as the density of cells per mm^2^.

### Intestinal microflora

The samples of cecum and ileum content (one bird from each replicate) for microbial analysis were collected by pressing the outer wall of the cut cecum and ileum to push its content into a sterile glass bottle. One gram of cecum and ileum content was blended in 9 mL of sterilized physiological saline solution and homogenized using a mixing bag. Subsequently, these suspensions were serially diluted from 10^−1^ to 10^−6^ using sterilized physiological saline solution. Duplicate plates per bird sample were inoculated with 100 μL of suspension and anaerobically incubated at 37°C.

Bacterial Culture and counts: Lactobacilli were anaerobically assayed using lactobacilli MRS agar (Fisher Scientific, Ottawa, Ontario, Canada) and incubated at 37°C for 48 h. Bifidobacteria counts were performed using Wilkins-Chalgren agar (Oxoid, Nepean, Ontario, Canada) supplemented with glacial acetic acid (1 mL/L) and mupirocin (100 mg/L) extracted from antimicrobial discs (Oxoid, UK). *Escherichia coli* bacteria were cultured on MacConkey’s agar medium (Oxoid, UK). The numbers of colony-forming units from duplicate plates per bird sample were averaged and the results were expressed as log colony-forming units per gram of intestinal content.

### Incidence of ascites

The incidence of ascites was determined using both the ascites mortality data tallied throughout the study along with the incidence of ascites present on the last day of the experiment. A RV/total ventricle ratio (RV/TV) >0.27 was used as a cutoff point for determining the ascites status of the birds. Chickens with an RV/TV ratio >0.27 were considered ascitic [17].

### Statistical analysis

Mean values generated from all individual data were statistically analyzed by a one-factor variance analysis using the GLM procedure of the SAS Institute (Cary, NC, USA). They were then compared using Tukey’s multiple range test at p<0.05 if the main effect between any groups was found to be significant. Person’s Chi-square test was used for the statistical evaluation of the incidence of ascites between treatment and control groups, if the main effects were significant at p<0.05.

## Results

### Blood parameters

Table-2 shows the effects of *A. niger* on the PCV, RBC, WBC, and Hb of chickens on day 42. The PCV values of Groups A3 and A5 were higher than in Groups A1, and the control (p<0.05). At the same time, Groups A4 showed higher PVC values than the control group (p<0.05). Groups A1, A2, A3, and A4 displayed higher (p<0.05) numbers of RBCs compared to C. The WBC counts of all groups were not significantly different. Groups A3, A4, and A5 revealed a higher value (p<0.05) than all the other groups (p>0.05) regarding the Hb values; Group A2 was significantly different from Groups A1 and C.

**Table 2:** The effect of the *Aspergillus niger* on the blood parameters of broiler chickens maintained under hypoxic conditions for a period of 42 days.

Treatments	Packed cell volume (%)	Red blood cell (10^6^×Cell/mm^3^)	White blood cell (10^3^×Cell/mm^3^)	Hemoglobin (%)
C	47.61c	3.21^b^	23.41	104.52^c^
A1	50.73^bc^	3.82^a^	24.32	106.51^c^
A2	50.22^bc^	3.85^a^	23.81	113.64^b^
A3	55.71^a^	3.95^a^	23.64	125.92^a^
A4	52.14^ab^	3.97^a^	24.15	127.61^a^
A5	56.23^a^	3.57^ab^	24.16	122.13^a^
SEM	1.96	0.125	0.897	4.080
p	0.005	0.031	0.426	0.027

Means within columns with different superscript letters are different per p<0.05

### Serum lipid profiles

Table-3 shows the effects of *A. niger* on total CH, TG, HDL, and LDL level. The serum CH value was significantly lower (p<0.05) in Groups A2, A3, A4, and A5 when compared to the control group. In particular, a lower value was observed in Group A3 and the differences were statistically significant compared to Groups A1 and C (p<0.05). Lower serum TG levels were observed in Groups A3, A4, and A5, this being significantly different (p<0.05) compared to the control. Regarding serum HDL levels, lower values were observed in Groups A1, A3, and A4, this value being significantly different than those observed for the control group (p<0.05). The LDL serum level was significantly lower (p<0.05) in all groups fed *A. niger* compared to the control group, and no difference (p>0.05) was observed between the experimental groups.

**Table 3:** The effect of the *Aspergillus niger* on serum lipid profiles of broiler chickens maintained under hypoxic conditions for a period of 42 days.

Treatments	CH (mg/dl)	Triglycerides (mg/dl)	High-density lipoproteins (mg/dl)	Low-density lipoprotein (mg/dl)
C	187.23^a^	67.32^a^	92.68^a^	92.62^a^
A1	182.65^ab^	64.64^ab^	85.55^b^	85.54^b^
A2	177.97^bc^	63.81^ab^	88.13^ab^	86.33^b^
A3	173.33^c^	61.43^b^	85.26^b^	87.64^b^
A4	175.54^bc^	61.34^b^	85.63^b^	86.55^b^
A5	176.67^bc^	62.87^b^	87.84^ab^	87.28^b^
SEM	6.191	2.352	3.307	3.461
p	0.042	0.034	0.026	0.018

Mean values within columns with different superscript letters are different per p<0.05

### Intestinal microflora

Table-4 shows the effects of dietary *A. niger* treatments on the intestinal microbial composition of birds at days 42. The dietary administration of *A. niger* significantly decreased (p<0.05) the concentration of *E. coli* in the cecum compared to the control groups and in the ileum compared to Groups A1 and control. The concentration of *Bifidobacterium* in the cecum significantly was increased (p<0.05) in Groups A2, A3, A4, and A5 compared to the control group. The concentration of *Bifidobacterium* in the ileum was increased (p<0.05) in Groups A3, A4, and A5 compared to Groups A1, A2, and control. The dietary administration of *A. niger* significantly increased (p<0.05) the concentration of *Lactobacillus* in the cecum. *Lactobacillus* counts in the ileum were significantly increased in Groups A2, A3, A4, and A5, compared to the control group, and the peak number appeared in Group A3 (p<0.05).

**Table 4:** The effect of food supplementation with *Aspergillus niger* on the number of intestinal microbial (log 10 cfu/g) located in broilers on day 42.

Treatments	Cecum			Ileum		
	
	*Escherichia coli*	*Bifidobacterium*	*Lactobacillus*	*Escherichia coli*	*Bifidobacterium*	*Lactobacillus*
C	8.89^a^	7.45±0.56^b^	7.86±0.37^b^	7.51^a^	6.06^b^	7.33±0.91^c^
A1	8.46^b^	8.23±0.33^ab^	8.85±0.54^a^	7.35^a^	6.26^b^	8.85±0.58^b^
A2	7.23^bc^	8.56±0.67^a^	9.32±0.76^a^	6.12^b^	6.24^b^	8.93±0.39^b^
A3	7.13^c^	8.69±0.56^a^	9.47±0.91^a^	6.02^b^	7.39^a^	9.15±0.69^a^
A4	7.25^bc^	8.65±0.76^a^	9.29±0.83^a^	6.06^b^	7.26^a^	9.09±0.86^ab^
A5	7.31^bc^	8.57±0.62^a^	9.36±0.76^a^	6.09^b^	7.32^a^	9.06±0.90^ab^
SEM	0.219	0.238	0.284	0.221	0.244	0.201
p	0.018	0.022	0.034	0.042	0.029	0.011

### Intestinal morphology and the incidence of ascites

Table-5 shows the effects of dietary addition of *A. niger* on the height of intestinal villus and depth of crypt in broiler chickens on day 42. Groups A3 and A4 had higher duodenum and ileum villi compared to the other groups (p<0.05). However, Groups A2, A3, and A4 had higher villi in the jejunum compared to the other groups (p<0.05). Duodenum crypt depth increased in Groups A3, A4, and A5 compared to the other groups (p<0.05). Jejunum and ileum crypt depth also increased significantly in Groups A2, A3, A4, and A5 compared to Groups A1 and control (p<0.05).

**Table 5:** The effect of the *Aspergillus niger* food supplementation on the height of intestinal villus and depth of crypt in 42 day-old broilers.

Treatments	The height of intestinal villus (μm)			The depth of crypt (μm)		
	
	Duodenum	Jejunum	Ileum	Duodenum	Jejunum	Ileum
C	1943.6^b^	1351.92^b^	731.53^b^	158.91^b^	149.88^b^	113.81^b^
A1	1936.8^b^	1345.62^b^	732.32^b^	165.35^b^	156.22^b^	120.15^b^
A2	1941.4^b^	1465.34^a^	723.44^b^	167.23^b^	192.49^a^	152.32^a^
A3	1982.6^a^	1456.76^a^	778.68^a^	206.78^a^	195.71^a^	155.67^a^
A4	1984.5^a^	1472.33^a^	773.26^a^	188.42^a^	192.14^a^	153.24^a^
A5	1928.4^b^	1360.57^b^	735.75^b^	191.69^a^	194.38^a^	154.61^a^
SEM	40.636	51.714	26.952	6.531	6.054	5.197
p	0.041	0.029	0.034	0.014	0.011	0.016

Means within columns with different superscript capital letters at same day are different (p<0.05)

Table-6 shows the effects of dietary addition of *A. niger* on the density of goblet cells in the intestine and on the incidence of ascites. Goblet cell density in the duodenum, jejunum, and ileum was highest in Groups A3, A4, and A5 compared to Groups A1 and control (p<0.05). Similarly, the difference in goblet cell density in Groups A1 and A2, compared to the control group, was significant (p<0.05).

**Table 6:** The effect of the *Aspergillus niger* on the density of goblet cells in intestine and the incidence of ascites in broiler chickens maintained under hypoxic conditions for 42 days.

Treatments	Goblet cell (cells/villus)			Ascites incidence (%)

	Duodenum	Jejunum	Ileum	
C	59.84±11.1^c^	59.84±11.1^c^	58.75±18.2^c^	65.74±6.16^a^
A1	67.56±6.25^bc^	82.54±6.54^b^	70.32±7.25^b^	54.21±5.21^b^
A2	78.45±7.85^ab^	86.23±7.25^b^	89.12±8.76^b^	52.45±4.88^b^
A3	82.64±8.01^a^	97.26±9.64^a^	103.45±12.20^a^	53.83±6.12^b^
A4	90.35±8.87^a^	97.56±8.79^a^	98.68±10.71^a^	51.26±5.32^b^
A5	87.32±7.56^a^	96.33±9.27^a^	101.34±11.24^a^	52.92±5.88^b^
SEM	3.376	3.577	4.651	2.283
p	0.018	0.011	0.013	0.037

Means within columns with different superscript capital letters at same day are different (p<0.05)

It was clear that all groups fed with *A. niger* showed significantly lower values compared to the control (p<0.05) regarding the incidence of ascites in broilers. Interestingly, no differences were observed between any of the experimental Groups A1-A5 (p>0.05).

## Discussion

Blood parameters (PCV, RBC, WBC, and Hb) were positively affected by the addition of *A. niger* to the diet of broiler chickens in this study. These results are in agreement with those observed by AI-Kassie *et al*. [5], who indicated that probiotic supplementation increased Hb values. It is reasonable to surmise that broilers have considerable difficulty meeting the oxygen demand required to maintain healthy tissue when hypoxia occurs. The addition of probiotics added to their feed seems to enhance growth performance by improving blood oxygen saturation, RBC production (partially immature), and Hb content [17]. Yet, Alkhalf *et al*. [18] found that probiotic diet supplementation did not cause significant increases in the erythrocyte count, Hb concentration, or hematocrit values of broilers. This could potentially be explained by the characteristics (type and number of species) of the probiotics employed in the different treatment regimes.

Our findings showed that the supplementation of broiler diets with *A. niger* significantly affected serum lipid profiles by decreasing serum CH, TG, HDL, and LDL levels. These results confirmed the findings of Al-Kassie *et al*. [5]. They suggested that there is a significant reduction in serum total CH levels in broilers fed with *A. niger*. Similar results have also been described by Yoon *et al*. [19]. According to these authors, hypocholesterolemia in broiler chickens was positively affected by the supplementation of ß-fructans from chicory as a source of probiotics. Furthermore, Zhu *et al*. [20], Saleh *et al*. [21], Al-Kassie *et al*. [5], and Saleh *et al*. [22] all agree that the reduction of serum CH levels could be due to the CH assimilation by *Lactobacillus*. Probiotic and prebiotic diet supplementation might enhance the lactobacilli count as well as the reduction of pH in the intestinal tract in this perspective.

The beneficial effects of probiotics on host indigenous microflora are well known [23]. This study indicates that the dietary administration of 1.0% and 1.25% of *A. niger* significantly decreased the concentration of *E. coli* in the cecum, while increasing the concentration of *Bifidobacterium* and *Lactobacillus* both in the cecum and ileum. Ohimain and Ofongo [23] reported a significant variation in relative amounts of bifidobacteria and lactobacilli in the intestinal content between the treatment groups, with generally more bifidobacteria produced with increased probiotic content.

Histological evaluation of the intestinal morphology has become a valuable tool in the assessment of developmental changes under different dietary conditions [24,25].

Increasing the villus height suggests an increased surface area capable of greater absorption of available nutrients. The villi play a crucial role in the digestion and absorption processes of the small intestine, as they increase the surface area and are the first to make contact with nutrients within the lumen [25]. Our observations demonstrated that a diet supplemented with 1.0% *A. niger* increased villi height in the duodenum, jejunum, and ileum of chickens. Several researchers have reported similar results. In one study, Awad *et al*. [26] found that a 0.1% probiotic supplementation appeared to increase villi heights in the duodenum and ileum. Awad *et al*. [27] described these alterations in the mucosal architecture in terms of increased ileal villus height in birds fed with a 0.1% symbiotic supplemented diet. Moreover, Pelicano *et al*. [28] showed that the addition of 0.1% probiotics to the diet resulted in longer jejunal and ileum villi in chickens. Furthermore, an increase in the villus length and width of the duodenum and ileum of broiler chickens treated with 0.1% prebiotic was reported by Sayrafi *et al*. [29].

Schwarz *et al*. [30] did not observe differences in crypt depth of the jejunum between the control group and the birds receiving diets containing *Bacillus subtilis*, but higher crypt depth was determined when probiotics based on *Lactobacillus fermentum* were used. Pelicano *et al*. [28] did not see differences in crypt depth in the ileum between the control groups and the groups receiving diets containing probiotics. This study demonstrated that the addition of 1.0% *A. niger* to the diet increased crypt depth in the duodenum, jejunum, and ileum of chicken on days 21. These findings corroborate the results regarding crypt depth described by Pelicano *et al*. [28] when probiotics were added to the diet.

Although Mair *et al*. [31] found that the density of acidic goblet cells in jejunal villi significantly decreased with the application of prebiotics to piglet diets, the current study demonstrated that the number of goblet cells in the duodenum, jejunum, and ileum increased by feeding 0.5%-1.5% *A. niger* to chickens. The improvement in crypt depth and density of goblet cells observed in probiotic-treated birds may be due to the rapid crypt maturation to ensure an adequate epithelium turnover rate, thus compensating for the losses [28].

Ascites syndrome in broilers is attributed to a higher metabolic burden which may lead to metabolic hypoxia or low efficiency of oxygen utilization. It is particularly severe when animals are raised in high altitude environments [16]. Our data indicate that the reduction of ascites incidence may be due to the probiotic treatment which increases the level of several blood parameters (RBC and Hb, overall). To this end, Solis de los Santos *et al*. [16] reported that broilers have difficulties in fulfilling tissue demands for oxygen in the case of hypoxia; thus, they exhibit a decrease in blood saturation and an increase in RBC production (partially immature). Simultaneously, a reduction in Hb content in RBCs might potentiate hypoxemia and the aggravation of ascites.

## Conclusion

Our results indicate that the inclusion of *A. niger* in diets of broilers, at a ratio ranging between 1.0% and 1.25%, enhances growth performance, blood parameters, intestinal morphology, and intestinal microflora. A reduction in serum CH level, *E. coli* in intestine, and ascites incidence was also observed. Collectively, these findings suggest that *A. niger* improves the resistance of chickens to hypoxic conditions. Moreover, the employment of probiotics is a viable alternative to the use of antibiotics in the agricultural production of broiler chickens. It represents an important step not only in increasing the environmental sustainability of broiler production but it is also a way of enhancing consumer safety. It is with this concern that we suggest further investigation into the interactions between the use of prebiotics and probiotics in birds raised under hypoxic conditions.

## Authors’ Contributions

HL: He is a master student who did most part of the research, contributed in, data collection, analysis, and manuscript. BD: Designed the research, method, data collection, and manuscript. LC: Data collection, analysis, and manuscript drafting. ZZ: Directed in method and data collection. HH: Directed in method and data collection. JW: Directed in method and data collection. XW: Directed in method and data collection. LZ: Directed in method and data collection. XN: Directed in method and data collection. BF: Designed the research, method, data collection, and manuscript. All authors read and approved the final manuscript.
